# Excitability of Anterior Horn Cells Following Repetitive Knee Joint Movements at Different Speeds

**DOI:** 10.7759/cureus.90618

**Published:** 2025-08-20

**Authors:** Masataka Kurobe, Yuki Takahashi, Naoki Kado, Toshiaki Suzuki

**Affiliations:** 1 Graduate School of Health Sciences, Graduate School of Kansai University of Health Sciences, Osaka, JPN; 2 Department of Physical Therapy, Kobe College of Rehabilitation and Health, Kobe, JPN

**Keywords:** f-wave, rehabilitation, repetitive movement, speed, vastus lateralis muscle

## Abstract

Introduction

F-waves are compound muscle action potentials generated by the antidromic activation of alpha motor neuron axons and the backfiring of anterior horn cells in response to electrical stimulation. Several F-wave parameters are used to assess the state of alpha motor neurons. Previous studies have demonstrated that repetitive thumb movements can decrease the F/M amplitude ratio in the abductor pollicis brevis muscle of healthy individuals. This suggests that repetitive movements may be a practical and accessible self-training method for alleviating muscle hypertonia. However, it remains unclear whether similar effects occur in the quadriceps femoris muscle following repetitive knee joint movement. Furthermore, the speed of movement may influence F-wave responses. This study aimed to investigate the effects of repetitive knee joint movements at different speeds on the excitability of anterior horn cells, as assessed by F-waves recorded from the vastus lateralis (VL) muscle before and after the movements.

Methods

Eighteen healthy adults participated in the study. F-waves were recorded from the VL muscle before, immediately after, and four and eight minutes after each task. In the 1 Hz trial, participants performed repetitive knee joint flexion and extension in synchrony with a 1 Hz auditory cue; in the 2 Hz trial, they moved in time with a 2 Hz cue. In the control trial, participants remained at rest. For F-wave recordings, the cathode of the stimulation electrode was placed at the distal one-fifth point along the line connecting the greater trochanter and the superolateral margin of the patella, with the anode placed at the distal one-fifth point along the line connecting the greater trochanter and the lateral joint space of the knee. The cathode of the recording electrode was positioned at the midpoint between the cathode of the stimulating electrode and the superior lateral border of the patella, and the anode was placed at the patella. The ground electrode was positioned on the anterior surface of the lower leg. Electrical stimulation was delivered at 1.2 times the intensity required to evoke the maximal M-wave amplitude, with a duration of 0.2 ms, a frequency of 0.3 Hz, and a number of stimuli of 60. The bandwidth filter was set between 20 and 3,000 Hz. Outcome measures included M-wave amplitude, F-wave persistence, and the F/M amplitude ratio. Since the Shapiro-Wilk test indicated non-normality, an aligned rank transformation was applied, followed by two-way repeated-measures ANOVA.

Results

There were no significant changes in the outcomes before or after repetitive knee movements. Additionally, movement speed did not affect the outcomes.

Conclusions

Unlike a previous report showing decreased excitability of anterior horn cells that innervate the abductor pollicis brevis following repetitive thumb movements, our study found no changes in VL excitability after repetitive knee movements performed at different speeds. These results imply that the modulation of anterior horn cell excitability by repetitive movements depends on the body part involved.

## Introduction

Muscle hypertonia is a common symptom of central nervous system disorders. In the lower limbs, hypertonia reduces walking speed and limits mobility [1]. Hypertonia is caused by abnormal excitability of alpha motor neurons and other components of the motor control system or increased muscle stiffness. Therefore, suppressing the excitability of alpha motor neurons is considered one approach to improving hypertonia; however, the specific self-training methods for achieving this remain unclear.

F-waves are compound muscle action potentials elicited by the antidromic activation of alpha motor neuron axons and the backfiring of anterior horn cells upon electrical stimulation. Several F-wave parameters are used to assess the state of alpha motor neurons: F-wave persistence reflects the excitability of the alpha motor neuron pool, the F/M amplitude ratio indicates the size of the backfiring motor units, and F-wave latency represents nerve conduction velocity [2]. Previous studies have shown that repetitive thumb movements can reduce the F/M amplitude ratio in the abductor pollicis brevis muscle in healthy individuals [3]. This suggests that repetitive movements may be a practical and accessible self-training method for alleviating muscle hypertonia. However, previous research has primarily focused on changes in central nervous system excitability following repetitive upper limb movements, despite known differences in neural responses between upper and lower limbs [4]. Thus, the effects of repetitive lower limb movements on the excitability of the central nervous system, particularly at the spinal level, remain unclear. Moreover, individuals with hypertonia may have difficulty performing rapid repetitive movements due to increased muscle tone. Therefore, it is necessary to determine whether F-wave parameters can be modulated by repetitive movements performed at slower speeds.

We consider it essential to investigate how to suppress the excitability of anterior horn cells in healthy individuals as a first step toward applying this approach to the treatment of individuals with central nervous system disorders. This study aims to investigate the effects of repetitive knee joint movements at different speeds on the excitability of anterior horn cells, as assessed by F-waves recorded from the vastus lateralis (VL) muscle before and after the movements. The hypothesis is that F-wave parameters decrease following repetitive movements, regardless of speed. Clarifying these effects may contribute to the development of accessible and effective self-training methods aimed at improving daily functioning in individuals with muscle hypertonia.

## Materials and methods

Participants

Eighteen healthy adults with their dominant foot on the right side (12 men and six women; mean age, 23.7 ± 7.2 years; mean height, 167.1 ± 9.3 cm; mean weight, 62.2 ± 10.1 kg; mean BMI, 22.2 ± 2.6 kg/m²) participated in the study. This study is the first to investigate the effects of repetitive lower limb movements on the excitability of anterior horn cells. As previous studies have typically involved several dozen participants, the sample size in the present study was determined accordingly [3,5-8]. Participants were recruited from the Kobe College of Rehabilitation and Health. Only individuals in generally good health were included, while those with a history or presence of neurological or orthopedic diseases were excluded.

Experimental design

Previous studies have shown that central nervous system excitability decreases following repetitive upper limb movements at 1 Hz or 2 Hz [3,5,6]. In the present study, F-waves were recorded before and after performing repetitive lower limb movements at 1 Hz, 2 Hz, or no movement. In the 1 Hz trial, after preparation, participants alternated between standing and sitting positions for one minute each. To familiarize participants with the sensation of electrical stimulation during F-wave recording, 60 preliminary stimuli were delivered. The purpose of these procedures was to standardize participants’ physiological condition before F-wave recording. F-waves were then recorded from the VL at rest. Participants subsequently performed the 1 Hz motor task. Following the motor task, F-waves were recorded three additional times in a resting state: immediately after the task, four minutes after, and eight minutes after. The reason for this is that, when recording F-waves from the VL, the stimulation frequency should be lowered to reduce pain caused by electrical stimulation [9]. Furthermore, since F-wave persistence is low at the VL, 60 stimulations were applied, which took about 200 seconds to record. The same procedure was followed for the 2 Hz trial, with participants performing the 2 Hz motor task. In the control trial, recordings were conducted without any motor task. Prerecorded instructions were displayed on a monitor to provide consistent guidance for all participants throughout the experiment. The order of the trials was randomized using a computer-generated random number sequence. A minimum 15-minute break was provided between trials to minimize carryover effects.

F-wave measurements

F-waves were recorded using a Viking Quest version 7.5 (Nicolet Biomedical Inc., Madison, WI, USA). Cup electrodes with a diameter of 10 mm were used as the cathode of the stimulating electrode, as well as the cathode of the recording electrode, the anode of the recording electrode, and the ground electrodes. A plate electrode with a diameter of 30 mm was used as the anode of the stimulating electrode.

**Figure 1:**
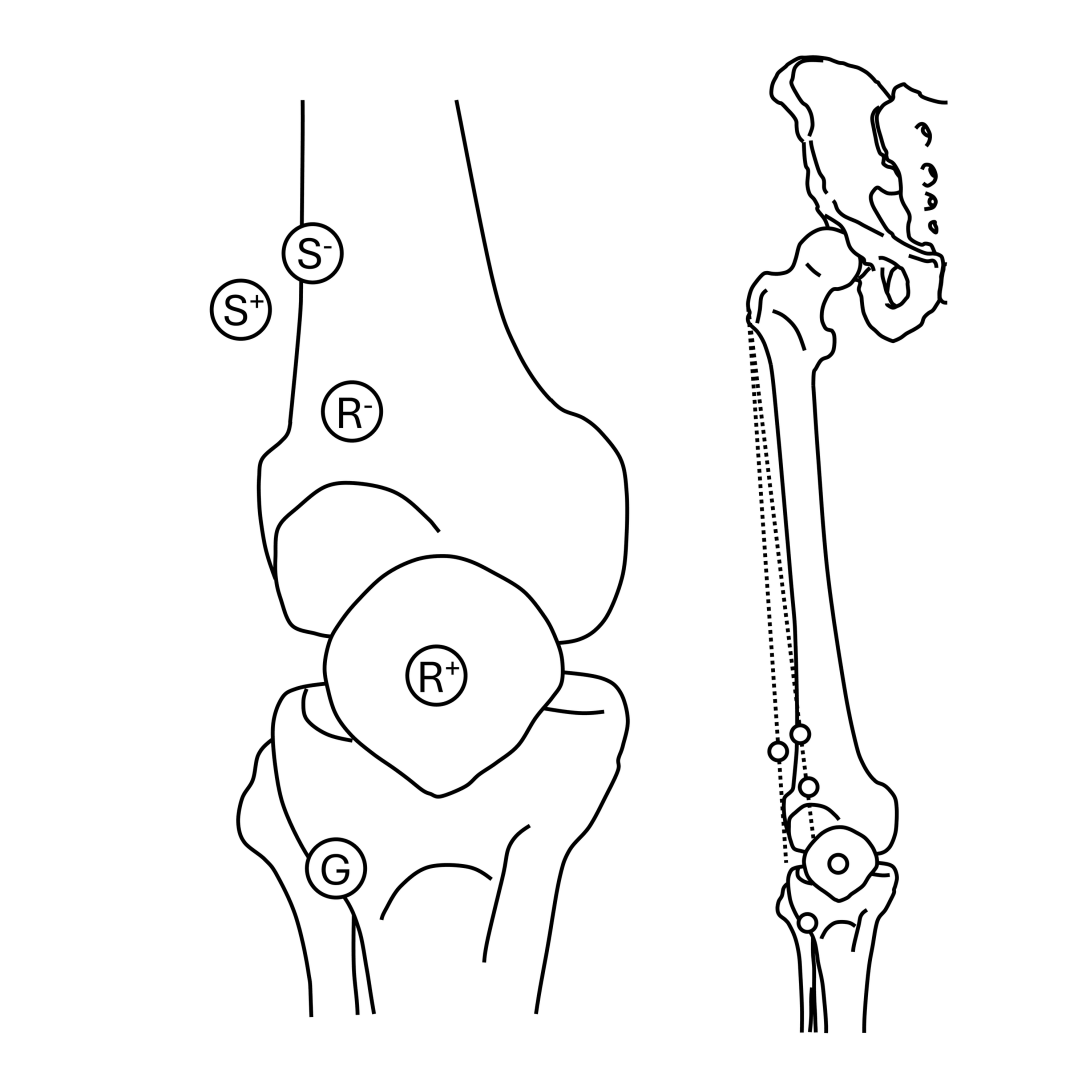
Position of the electrodes The stimulating electrode cathode (S-), the stimulating electrode anode (S+), the recording electrode cathode (R-), the recording electrode anode (R+), and the ground electrode (G).

Prior to electrode application, the participant’s skin was wiped with alcohol, and an abrasive cream was applied and gently rubbed on the electrode attachment site. Using tape, the stimulating electrode cathode was applied to the physiological motor point of the distal VL. The physiological motor point is defined as the location that can induce muscle contraction even with low-intensity stimulation [10]. Stimulation of this area allows F-waves to be recorded even with weak stimuli. The motor point of the distal VL is located approximately at the distal one-fifth of the line extending from the greater trochanter to the superior lateral border of the patella [11]. The examiner adjusted the stimulating electrode cathode placement within this area to determine the site that elicited the highest M-wave amplitude in response to weak stimuli. The stimulating electrode anode was positioned at the distal one-fifth of a line extending from the right greater trochanter to the lateral space of the right knee joint. The recording electrode was attached to the distal portion of the VL to delay the onset of the F-wave and minimize its overlap with the M-wave. In detail, it was positioned between the cathode of the stimulating electrode and the superior lateral border of the right patella. The anode of the recording electrode was attached to the right patella, and the ground electrode was attached to the anterior surface of the right lower leg (Figure 1).

Stimulation intensity was determined as follows. First, the stimulus intensity was increased in 5 mA increments until the maximum M-wave amplitude was obtained. Next, it was decreased in 1 mA steps to identify the minimal stimulus intensity that still elicited the maximal M-wave. Finally, this stimulus intensity was multiplied by 1.2 and used as the stimulation intensity for F-wave recording. The stimulus duration was set at 0.2 ms, the frequency at 0.3 Hz, and the number of stimuli at 60. The bandwidth filter was set between 20 Hz and 3,000 Hz.

During the experimental session, participants were instructed to remain relaxed in a seated position with their right knee joint fixed at a 45° flexion. The room temperature was maintained at 25°C to prevent changes in nerve conduction velocity due to fluctuations in body temperature.

Motor task

**Figure 2:**
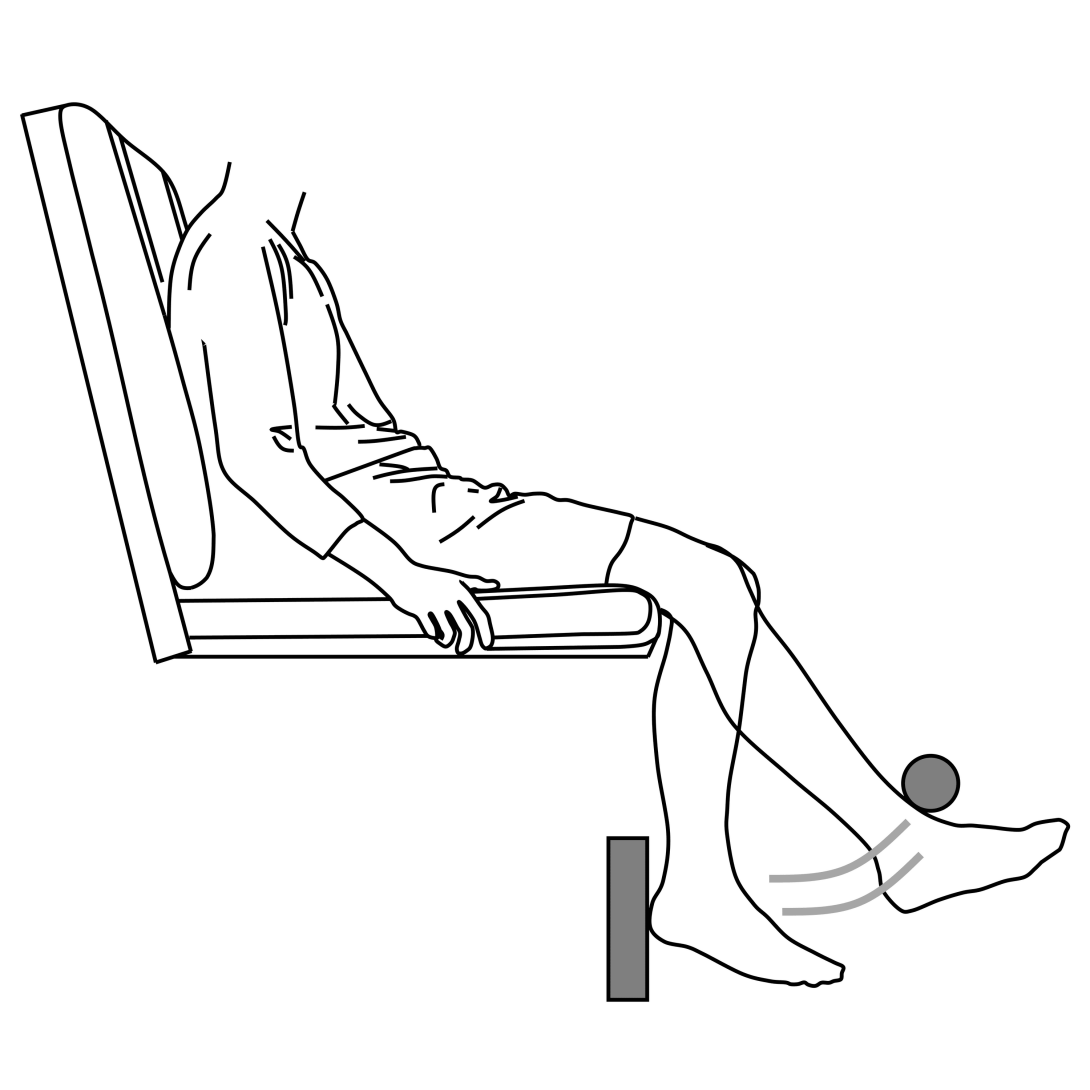
Situation of the motor task

The participants were seated with both legs suspended off the ground. A bar was positioned in front of the right leg, and a board was placed behind it to define the range of motion. The anterior bar was set to make contact with the front of the ankle joint when the right knee joint was extended to 45°, while the posterior board was positioned to allow the heel to touch it when the right knee joint was flexed to 90° (Figure 2).

The task was performed without resistance, similar to the previous study [3,8]. The participants repeated flexion and extension of the knee joint in synchrony with a metronome. The metronome frequency was set to either 1 Hz or 2 Hz, with each task lasting 120 seconds. During the 1 Hz motor task, the knee joint extended to the anterior bar in 1 second and flexed to the posterior board in another 1 second. In contrast, the 2 Hz motor task required knee joint extension and flexion to be completed in 0.5 seconds each.

Data analysis

Viking Quest 7.5 does not have a function to correct the baseline of the F-wave, and the F-wave amplitude may be underestimated because the baseline of the F-wave is oblique due to the M-wave tail. Therefore, the data were transferred to Viking Quest version 20.0 (Natus Medical Inc., Middleton, WI, USA), and the baseline of the F-wave was corrected by the “Signal Enhancer function.” Then, blinded to which trial's waveforms, the M- and F-waves were analyzed.

The M-wave amplitude was defined as the peak-to-peak amplitude of the M-wave recorded from the 31st stimulus in each trial. Previous studies have considered a minimum amplitude of 20, 30, or 40 μV for identifying F-waves [12-14]. In the present study, any wave with an amplitude higher than 30 μV that occurred after the M-wave was defined as an F-wave. F-wave persistence was calculated as the number of F-waves divided by the number of stimuli and was expressed as a percentage. The F/M amplitude ratio was calculated by dividing the mean F-wave amplitude by the M-wave amplitude and expressed as a percentage.

Statistical analysis was performed using IBM SPSS Statistics for Windows, Version 20.0 (Released 2017; IBM Corp., Armonk, NY, USA). The Shapiro-Wilk test was used to assess the normality of data. For non-normally distributed data, the aligned rank transformation was applied. Repeated measures two-way ANOVA was conducted for M-wave amplitude, F/M amplitude ratio, and F-wave persistence at four time points: before the task, immediately after, four minutes after, and eight minutes after. Time and motor task were included as factors. Generalized η² was calculated as a measure of effect size. The significance level of p < 0.05 was considered significant.

## Results

As all outcome measures were not normally distributed, an aligned rank transform was applied prior to conducting two-way repeated measures ANOVA.

M-wave amplitude (mV)

No significant main effect of time was observed (F = 0.010, p = 0.999, η²G < 0.001), nor was there a significant main effect of task (F = 0.062, p = 0.940, η²G < 0.001). In addition, no significant interaction between time and task was found (F = 2.233, p = 0.111, η²G = 0.001) (Table 1).

**Table 1:** M-wave amplitude (mV) of each trial Pre: before the motor task; Post0: immediately after the motor task; Post4: four minutes after the motor task; Post8: eight minutes after the motor task

Trial	Timepoint	Median	IQR	Range
Control trial	Pre	8.85	5.20-10.30	3.11-11.71
	Post0	8.88	5.15-10.54	3.27-11.78
	Post4	8.86	5.47-10.48	3.14-12.08
	Post8	8.81	5.41-10.78	3.16-12.14
1 Hz trial	Pre	7.63	4.53-9.24	2.54-12.19
	Post0	7.46	4.77-9.24	2.76-13.00
	Post4	7.71	4.71-9.20	2.94-12.79
	Post8	7.76	4.69-9.96	3.08-12.66
2 Hz trial	Pre	7.88	5.61-9.18	2.75-13.09
	Post0	8.52	5.60-9.28	2.62-12.89
	Post4	8.32	5.29-9.12	2.72-13.32
	Post8	8.29	5.26-9.73	2.88-13.49

F-wave persistence (%)

Similarly, no significant main effects were found for time (F = 0.397, p = 0.757, η²G = 0.002) or task (F = 0.969, p = 0.401, η²G = 0.007). The interaction between time and task was also not significant (F = 1.124, p = 0.404, η²G = 0.004) (Table 2).

**Table 2:** F-wave persistence (%) of each trial Pre: before the motor task; Post0: immediately after the motor task; Post4: four minutes after the motor task; Post8: eight minutes after the motor task

Trial	Timepoint	Median	IQR	Range
Control trial	Pre	15.83	9.17-30.00	1.67-55.00
	Post0	15.83	10.00-36.25	1.67-56.67
	Post4	12.50	5.42-24.58	3.33-43.33
	Post8	10.00	8.33-27.92	1.67-45.00
1 Hz trial	Pre	16.67	10.42-36.25	1.67-76.67
	Post0	24.17	8.33-31.67	0.00-81.67
	Post4	22.50	6.67-39.17	0.00-78.33
	Post8	15.83	7.50-29.58	0.00-70.00
2 Hz trial	Pre	18.33	7.08-30.42	0.00-73.33
	Post0	22.50	3.75-30.83	0.00-85.00
	Post4	14.17	7.08-29.58	0.00-73.33
	Post8	10.83	3.33-35.42	0.00-70.00

F/M amplitude ratio (%)

There were no significant main effects of task (F = 2.043, p = 0.151, η² = 0.005) or time (F = 1.849, p = 0.189, η² = 0.012). The interaction between task and time was also not significant (F = 0.241, p = 0.954, η² < 0.001) (Table 3).

**Table 3:** F/M amplitude ratio (%) of each trial Pre: before the motor task; Post0: immediately after the motor task; Post4: four minutes after the motor task; Post8: eight minutes after the motor task

Trial	Timepoint	Median	IQR	Range
Control trial	Pre	0.26	0.07-0.49	0.02-1.26
	Post0	0.24	0.11-0.39	0.01-1.28
	Post4	0.16	0.07-0.30	0.02-1.07
	Post8	0.18	0.12-0.35	0.01-0.73
1 Hz trial	Pre	0.37	0.11-0.55	0.02-1.41
	Post0	0.33	0.12-0.68	0.00-1.31
	Post4	0.36	0.08-0.70	0.00-1.21
	Post8	0.32	0.09-0.46	0.00-0.98
2 Hz trial	Pre	0.20	0.09-0.46	0.00-1.55
	Post0	0.26	0.04-0.48	0.00-1.54
	Post4	0.14	0.08-0.38	0.00-1.34
	Post8	0.19	0.03-0.36	0.00-1.29

## Discussion

This study investigated the effects of repetitive knee joint movements at different speeds on the excitability of anterior horn cells, as assessed by F-waves recorded from the VL before and after movement. We hypothesized that F-wave parameters would decrease after repetitive knee movements, regardless of speed. However, the results showed no significant changes in M-wave amplitude, F-wave persistence, or the F/M amplitude ratio under any condition. These findings suggest that repetitive knee joint movement does not alter the excitability of the VL or the anterior horn cells. To the best of our knowledge, this is the first study to investigate the effects of repetitive knee joint movement at varying speeds on muscle and anterior horn cell excitability.

The M-wave amplitude remained stable immediately after the task and up to eight minutes post-task, compared to pre-task values. This finding is consistent with previous reports showing no changes in M-wave amplitude in finger muscles after repetitive finger movements [3]. The stability of M-wave amplitude indicates that the positions of both stimulation and recording electrodes were consistent for most participants throughout the experiment. Minor variations in M-wave amplitude observed in some participants may have been due to slight differences in sitting posture. Additionally, previous research demonstrated an increase in M-wave amplitude after 48 maximal isometric contractions of the VL; however, the present study involved low-intensity, non-fatiguing movements without inducing muscle fatigue, which likely explains the absence of M-wave amplitude changes [15].

Similarly, no significant changes were observed in F-wave persistence or the F/M amplitude ratio, suggesting that the excitability of the anterior horn cells was not affected by the repetitive knee joint movement. Some studies investigating the effects of repetitive movement on neural excitability have evaluated motor-evoked potentials (MEPs) using transcranial magnetic stimulation to assess corticospinal excitability [6-8,16]. In contrast, only a few studies have utilized F-wave parameters as indicators of anterior horn cells’ excitability [3,5]. Moreover, these studies have focused on finger movements; no studies to date have examined the effects of repetitive knee joint movements on these parameters.

The speed and duration of motor tasks are known to influence corticospinal excitability after movements. For example, MEPs have been shown to decrease after movement at 2 Hz but not at 1 Hz [6]. In contrast, another study reported a greater reduction in MEP amplitude after tasks performed at half the maximum speed compared to those executed at maximum speed [7]. Additionally, a reduction in the F/M amplitude ratio has been observed after a 1 Hz task [3]. Due to these inconsistent findings regarding the effects of movement speed on the nervous system, the present study included both 1 Hz and 2 Hz tasks. Regarding task duration, previous studies have employed durations ranging from 10 to 600 seconds [3,7,17]. Miyaguchi et al. reported no significant differences in MEP amplitude changes between 120- and 360-second tasks [8]. In this study, we wanted to develop simple and practical training methods, so we set the task time to 120 seconds. Although both speed and duration were selected based on prior research, no significant changes in F-wave parameters were observed. These results suggest that the responsiveness of anterior horn cells may vary depending on where repetitive movements are performed in the body.

The possible mechanisms may involve the serotonergic system and the cerebral cortex. It has been suggested that two pathways, the raphe nucleus-spinal tract and the corticospinal tract, are involved in modulating anterior horn cell excitability following repetitive movements. Serotonin, synthesized in the raphe nuclei, is released via descending fibers that terminate on the dendrites of motor neurons in the spinal cord [18]. When serotonin is released in large amounts and 5-HT_1A_ receptors are activated, the excitability of motor neurons decreases [19,20]. Previous studies have shown that administration of selective serotonin reuptake inhibitors before maximal isometric contractions leads to reduced F-wave persistence post-exercise, suggesting the involvement of the raphe nucleus-spinal pathway in modulating spinal anterior horn cell excitability [21]. Despite these mechanisms, no significant changes in F-wave parameters were observed in this study. A likely reason is that high-intensity contractions are necessary to induce sufficient serotonin release to activate inhibitory mechanisms [21]. In the present study, the tasks involved low-intensity, non-fatiguing movements without additional loading, which may have been insufficient to trigger serotonin-mediated inhibition of motor neurons via 5-HT_1A_ receptors. Following isotonic contractions, the excitability of the corticospinal tract decreases due to the activation of GABAergic inhibitory mechanisms in the motor cortex [7]. However, anatomical studies indicate that the density of direct corticospinal projections to spinal motor neurons is lower in the proximal lower limb muscles than in the upper limbs [22,23]. Therefore, in this study, the GABA-mediated cortical inhibitory system may have exerted little influence on anterior horn cells via the corticospinal tract, which could account for the absence of changes in F-wave parameters after the knee joint movements.

Although repetitive movement has been considered a potential means of reducing muscle tone, the present findings suggest that such movement does not suppress the excitability of anterior horn cells innervating the VL. This implies that the effects of exercise on muscle tone may vary depending on the target muscle. However, different outcomes may be obtained under alternative movement conditions. For instance, it remains to be determined whether F-wave parameters would change after repeated knee joint movements under load.

The limitations of this study are that it was conducted in healthy participants; results may differ in patients with central nervous system diseases who have increased muscle tone [24]. In addition, the sample size was small. F-waves were recorded only from VL. Muscle activity during the task could not be recorded using surface electromyography. Furthermore, other neurological indicators such as MEP and H-reflex could not be recorded. Additionally, objective fatigue assessment was not performed in this study; however, participants did not report any fatigue.

## Conclusions

Unlike a previous report showing decreased excitability of anterior horn cells that innervate the abductor pollicis brevis following repetitive thumb movements, our study found no changes in VL excitability after repetitive knee movements performed at different speeds. These results imply that the modulation of anterior horn cell excitability by repetitive movements depends on the body part involved. Furthermore, under the present conditions, repetitive knee movements may not be an effective self-training method for alleviating muscle hypertonia.

## References

[1] Banky M, Ross H, Williams G, Kahn M (2025). The distribution and severity of lower-limb hypertonicity and spasticity differentially impacts walking speed in people with neurological injuries. Disabil Rehabil.

[2] Mesrati F, Vecchierini MF (2004). F-waves: neurophysiology and clinical value. Neurophysiol Clin.

[3] Kurobe M, Matsubara H, Suzuki T (2021). Excitability of anterior horn cells after periodic or discrete repetitive movements. Muscle Nerve.

[4] Carroll TJ, Baldwin ER, Collins DF, Zehr EP (2006). Corticospinal excitability is lower during rhythmic arm movement than during tonic contraction. J Neurophysiol.

[5] Zanette G, Bonato C, Polo A, Tinazzi M, Manganotti P, Fiaschi A (1995). Long-lasting depression of motor-evoked potentials to transcranial magnetic stimulation following exercise. Exp Brain Res.

[6] Bonato C, Zanette G, Fiaschi A, Rossini PM (2002). Activity-dependent modulation of synaptic transmission in the intact human motor cortex revealed with transcranial magnetic stimulation. Cereb Cortex.

[7] Teo WP, Rodrigues JP, Mastaglia FL, Thickbroom GW (2012). Post-exercise depression in corticomotor excitability after dynamic movement: a general property of fatiguing and non-fatiguing exercise. Exp Brain Res.

[8] Miyaguchi S, Kojima S, Kirimoto H, Tamaki H, Onishi H (2016). Do differences in levels, types, and duration of muscle contraction have an effect on the degree of post-exercise depression?. Front Hum Neurosci.

[9] Kurobe M, Suzuki T (2023). Pain reduction method in recording F-waves from the vastus lateralis muscle. Muscle Nerve.

[10] Knaflitz M, Merletti R, De Luca CJ (1990). Inference of motor unit recruitment order in voluntary and electrically elicited contractions. J Appl Physiol (1985).

[11] Botter A, Oprandi G, Lanfranco F, Allasia S, Maffiuletti NA, Minetto MA (2011). Atlas of the muscle motor points for the lower limb: implications for electrical stimulation procedures and electrode positioning. Eur J Appl Physiol.

[12] Weber F (1998). The diagnostic sensitivity of different F wave parameters. J Neurol Neurosurg Psychiatry.

[13] Zhou HH, Zhu C (2000). Comparison of isoflurane effects on motor evoked potential and F wave. Anesthesiology.

[14] Nobrega JA, Manzano GM, Monteagudo PT (2001). A comparison between different parameters in F-wave studies. Clin Neurophysiol.

[15] Rodriguez-Falces J, Place N (2017). Different recoveries of the first and second phases of the M-wave after intermittent maximal voluntary contractions. Eur J Appl Physiol.

[16] Avanzino L, Tacchino A, Abbruzzese G (2011). Recovery of motor performance deterioration induced by a demanding finger motor task does not follow cortical excitability dynamics. Neuroscience.

[17] Crupi D, Cruciata G, Moisello C (2013). Protracted exercise without overt neuromuscular fatigue influences cortical excitability. J Mot Behav.

[18] Alvarez FJ, Pearson JC, Harrington D, Dewey D, Torbeck L, Fyffe RE (1998). Distribution of 5-hydroxytryptamine-immunoreactive boutons on alpha-motoneurons in the lumbar spinal cord of adult cats. J Comp Neurol.

[19] Cotel F, Exley R, Cragg SJ, Perrier JF (2013). Serotonin spillover onto the axon initial segment of motoneurons induces central fatigue by inhibiting action potential initiation. Proc Natl Acad Sci U S A.

[20] Perrier JF, Rasmussen HB, Jørgensen LK, Berg RW (2017). Intense activity of the raphe spinal pathway depresses motor activity via a serotonin dependent mechanism. Front Neural Circuits.

[21] Kavanagh JJ, McFarland AJ, Taylor JL (2019). Enhanced availability of serotonin increases activation of unfatigued muscle but exacerbates central fatigue during prolonged sustained contractions. J Physiol.

[22] Weil A, Lassek A (1929). The quantitative distribution of the pyramidal tract in man. Arch Neurol Psychiatry.

[23] Eisner-Janowicz I, Chen B, Sangari S, Perez MA (2023). Corticospinal excitability across lower limb muscles in humans. J Neurophysiol.

[24] Scalia M, Borzuola R, Parrella M, Borriello G, Sica F, Monteleone F, Macaluso A (2025). Neuromuscular electrical stimulation reduces spinal excitability in multiple sclerosis patients with spasticity symptoms. Mult Scler Relat Disord.

